# Supplementary material for: Incidence and predictors of cardiovascular disease mortality and all-cause mortality in patients with type II diabetes with peripheral arterial disease
Source: PLoS One. 2025 May 16;20(5):e0322502. doi: 10.1371/journal.pone.0322502 (PMC12083800; doi:10.1371/journal.pone.0322502)
Supplement: S1 Table — (DOCX) [file pone.0322502.s001.docx]

| **Supplement Table 1.** Risk factors management ^a^ at baseline of participants with type 2 diabetes and peripheral arterial disease (PAD). (N=278) | | | | |
| --- | --- | --- | --- | --- |
| **Variables** | **Survivors** | **Non-CVD deaths** | **CVD deaths** | ***p-*value** |
| n | 178 | 42 | 58 |  |
|  |  |  |  |  |
| Glycemic control (HbA1c <7% or fasting blood sugar <130 mg/dL) | 61 (34.3) | 18 (42.9) | 29 (50.0) | .087 |
|  |  |  |  |  |
| Blood pressure control (<130/80 mmHg) | 59 (33.2) | 13 (31.0) | 20 (34.5) | .93 |
|  |  |  |  |  |
| Low-density lipoprotein cholesterol control (LDL-C) (<70 mg/dL) | 37 (20.8) | 8 (19.0) | 11 (19.0) | .94 |
|  |  |  |  |  |
|  |  |  |  |  |
| Data are presented as n (%).  ^a^ This criterion adheres to the 2019 ESC Guidelines, which provide recommendations for managing blood pressure, dyslipidemia, and glucose levels in patients with diabetes and established cardiovascular disease, a group categorized as having a high cardiovascular risk. | | | | |
