# Supplementary material for: Incidence and predictors of cardiovascular disease mortality and all-cause mortality in patients with type II diabetes with peripheral arterial disease
Source: PLoS One. 2025 May 16;20(5):e0322502. doi: 10.1371/journal.pone.0322502 (PMC12083800; doi:10.1371/journal.pone.0322502)
Supplement: Attachment — Submitted filename: Response to Reviewers_R1# PONE-D-24-50975.docx [file pone.0322502.s003.docx]

**RESPONSES TO REVIEWER'S COMMENTS**

**“Incidence and predictors of cardiovascular disease mortality and all-cause mortality in patients with type II diabetes with peripheral arterial disease**

**# PONE-D-24-50975”**

**Shukri AlSaif (Academic Editor)**

**Thank you for submitting your manuscript to PLOS ONE. After careful consideration, we feel that it has merit but does not fully meet PLOS ONE’s publication criteria as it currently stands. Therefore, we invite you to submit a revised version of the manuscript that addresses the points raised during the review process.**

Dear Dr. AlSaif

Thank you for giving us the opportunity to revise and re-submit our manuscript to PLOS ONE.

We extend our sincere gratitude to you and the reviewers for the detailed and constructive feedback. In response, we have made significant revisions to the manuscript as suggested. Please find below our detailed responses to your and the reviewers' comments. All pages refer to the revised manuscript. In the tracked changes version, revisions in response to the reviewers and editors' comments are highlighted in yellow.

We hope this revised version meets the journal's standards and is now suitable for publication. However, we are open to making any further revisions that may be necessary. We look forward to hearing from you again in due course.

Kind regards,

Amaraporn Rerkasem, on behalf of all authors

**REVIEWER #1**

**PADs are classical but uprising diseases and the predictors are more to be focused.**

Reply: We appreciate the thoughtful comments from the reviewers regarding our manuscript. We have addressed each point as follows:

**Major:**

**1) Why the recently diagnosed CI or CAD patients were excluded ?**

Reply: Thank you for this important question. Regarding the exclusion of recently diagnosed cerebrovascular and coronary artery disease patients, this methodological decision was made to establish a stable baseline population where measurements reflect steady-state disease rather than acute post-event changes. The 3-month exclusion window helps minimize the confounding effects of immediate post-event treatments and temporary changes in cardiovascular risk factors that typically occur after acute events. This approach allows for a more reliable assessment of PAD's natural progression and its relationship with future cardiovascular outcomes. We have clarified this in our Study Population Subsection under Material and Methods Section and added the specific 3-month timeframe, which was not explicitly stated in our original manuscript now read as follows:

*“The exclusion criteria were as follows: patients with a new diagnosis of cerebrovascular or coronary events within three months of enrollment to ensure baseline measurements reflected stable disease states rather than acute post-event changes. Individuals with stage III-IV malignancy or terminal cancer and a life expectancy less of than one year, as well as those with active HIV infection or AIDS-related vasculopathy were also excluded. Furthermore, patients requiring scheduled vascular surgery within the following six months —such as those with symptomatic abdominal aortic aneurysms or critical limb ischemia—were not included. Finally, those with systemic infections affecting the vasculature, such as fungal arteritis, were excluded from study.”*

**2) While we are researching natural course of the PAD, I think this part is absolutely important. Also, Table1 contains history of OMI, old CI, and TIA. If you are focusing only "future (newly complicated)" CI or CAD, the title need some arrangement.**

Reply: We appreciate this astute observation. Concerning the presence of historical cardiovascular events in Table 1 while excluding recent events, this reflects our intent to capture the real-world comorbidity burden in the PAD population while avoiding the confounding effects of acute disease states. Our current title appropriately reflects the study's focus on mortality outcomes in this population, regardless of their cardiovascular history. We have clarified the distinction between included historical events and excluded recent events in our Methods Section (in reply of question #1) and acknowledged in our limitations as follows:

*“The exclusion of patients with recent cardiovascular events (within 3 months of enrollment), though necessary for establishing a stable baseline population, may have underestimated the full spectrum of cardiovascular risk in PAD patients. ”*

**3) Do authors have any idea about medical treatment backgrounds? There are only about insulins and DPP4 (Table).**

Reply: Thank you for highlighting the importance of medical treatment backgrounds. We agree that cardiovascular medications known to affect mortality in PAD patients should be considered in our analysis. Following your comment, we conducted additional analyses incorporating key cardiovascular medications based on their clinical relevance, observed distribution differences between study groups, and established effects on mortality from literature. These included antithrombotic agents, statin therapy, and RAAS blockers (combined ACEI/ARBs) alongside diabetes medications.

This expanded analysis revealed insulin sensitizer therapy as a protective factor for all-cause mortality, while other previously identified risk factors (critical limb ischemia, being underweight, and chronic kidney disease) remained significant predictors. The predictors of cardiovascular disease-related death remained consistent with our original findings.

We have revised **Tables 4 and 5** to reflect these analyses and added a new discussion paragraph that provides a comprehensive understanding of how insulin sensitizer therapy influences mortality outcomes in our study population.

*“**Additionally, this study identified a protective effect of insulin-sensitizing hypoglycemic drugs on overall mortality, but not specifically on CVD mortality, in PAD patients. Insulin sensitizers, including thiazolidinediones and biguanides, reduce insulin resistance and may slow atherosclerosis progression [33]. While randomized controlled trials have shown that these drugs could reduce all-cause mortality and non-fatal cardiovascular events in T2DM patients [34], some studies have reported an increased risk of heart failure [35]. Research specifically examining mortality outcomes in T2DM patients with PAD using insulin sensitizers remains limited. Our findings suggest potential benefits of insulin sensitizers beyond glycemic control in this high-risk population, though further research is needed to confirm these effects.”*

**Minor:**

**1) ABI cannot stage severity of the PAD. (abstract part)**

Reply: Thank you for your astute observation regarding ABI and PAD staging. You are correct that ABI alone cannot stage PAD severity. We have revised the abstract to clarify that PAD severity was determined through both clinical assessment and ABI measurements.

*“These patients completed health questionnaires and underwent physical examinations including ankle-brachial index (ABI) measurements and clinical assessment to determine PAD severity.”*

**2) Check acronyms. DM or T2DM (better to unify), several acronyms are spelled out twice or more.**

Reply: Thank you for your comment. We have carefully reviewed all acronyms in the manuscript and ensured consistency in their usage. Specifically, we have standardized the use of **T2DM** throughout the text instead of alternating between **DM** and **T2DM**. Additionally, we have removed redundant definitions of acronyms that were spelled out more than once. We appreciate your suggestion, which has helped improve the clarity and consistency of our manuscript.

**3) PADs are classified as asymptomatic, intermittent claudication, and CLI. Is the rest pain included as CLI ? Personally, I want to know the difference between ulcer and rest pain.**

Reply: Thank you for this important question about PAD classification. In our study, we classified rest pain as part of critical limb ischemia (CLI) following the TASC II 2007 guidelines. This classification reflects that both rest pain and tissue loss represent advanced stages requiring similar management approaches, distinct from intermittent claudication and asymptomatic PAD. Additionally, given the relatively small number of events in our real-world data, combining these presentations into the CLI category enabled more robust statistical analysis while maintaining clinical relevance. We have expanded the definition of CLI alongside the TASC II reference in the introduction section to provide better clarity.

*“Critical limb ischemia (CLI), an advanced stage of PAD characterized by rest pain, ischemic ulcer, or gangrene [8], often results in amputation and death. [8]”*

**4) Any idea for the ADL ? (walking/chair-bound/bed-bound etc.)**

Reply: Thank you for raising this important point about ADL status. While ADL was not systematically collected at enrollment as it was not part of our primary objectives, we did find in nursing records that 268 participants were ambulatory while 10 were chair-bond and bed-bound, with bed-bound status mainly occurring in CLI patients. We acknowledge this as a study limitation in the revised manuscript, as functional status could be an important mortality predictor in PAD patients. Future studies should systematically assess ADL status to better understand its relationship with mortality in this population. A limitation statement in the Limitations Section of the manuscript, now read as follows:

*“Another significant limitation was the lack of systematic collection of activities of daily living (ADL) data at enrollment. Nursing records indicated that most participants (n=268) were ambulatory, while some arrived at the clinic using a wheelchair (n=4) or a stretcher (n=6), primarily those with CLI. However, the lack of comprehensive functional status data limited our ability to analyze ADL as a potential predictor of mortality.”*

**5) Any data for the hemodialysis patient ?**

Reply: Thank you for inquiring about hemodialysis patients. Our study included 6 patients (2.2%) on maintenance hemodialysis at baseline. While hemodialysis is known to be associated with increased CVD mortality risk, our small sample size precluded robust statistical analysis of this relationship. So, we assessed kidney function primarily through eGFR<60 ml/min/1.73m² to capture the broader spectrum of CKD in our cohort. We have added hemodialysis details to the second paragraph of the Results section after the CKD findings (in Table 1), which now reads:

*“Of the patients with CKD, 6 patients were on maintenance hemodialysis at baseline. Among these hemodialysis patients, 6 died during follow-up (3 from CVD-related causes, 3 from non-CVD causes).”*

**6) Any idea for the location of the PAD ? (iliac/femo-pop/BK)**

**Reply:** Thank you for your comment. Identifying the specific anatomical location of PAD lesions requires advanced angiographic imaging (e.g., DSA, CTA, MRA), which, per the European guideline 2019, is typically reserved for patients requiring revascularization. Non-invasive methods like physical examination and ABI measurements are insufficient for precise localization.

In our study, 90% (251 of 278) of patients presented with asymptomatic PAD or claudication, effectively managed with conservative treatments, and thus did not require angiographic evaluation. Among the 10% (27 of 278) with critical limb ischemia (CLI), angiographic evaluation was performed in 5 cases where revascularization was feasible. These lesions were primarily in the femoropopliteal and tibial segments, with iliac involvement noted in four cases. The remaining CLI patients were unsuitable for revascularization due to extensive necrosis, gangrene, or severe comorbidities, and angiographic evaluations were not performed.

To address this further, we revised the inclusion criteria and added a new paragraph in the Study Population subsection of the Methods section to clarify the rationale for the limited lesion location data. The updates are as follows:

***Inclusion criteria:*** *"…Inclusion criteria required a diagnosis of T2DM, age ≥45, and PAD confirmed by symptoms or an ankle-brachial index (ABI) ≤0.9. Imaging techniques, such as duplex ultrasound, digital subtraction angiography (DSA), computed tomography angiography (CTA), or magnetic resonance angiography (MRA) were used for confirmation in selected cases when clinically indicated. All participants were required to have been under hyperglycemic control for at least six months before enrollment. "*

***New paragraph:*** *"Due to the inclusion criteria and clinical characteristics of the study population, the anatomical location of PAD was not uniformly determined. Advanced imaging techniques (e.g., DSA, CTA, MRA) were reserved for cases where revascularization was clinically necessary, in line with European guidelines [11]. Initial assessments relied on physical examination, ABI measurements, and duplex ultrasonography, which are sufficient for diagnosing PAD but not for detailed anatomical localization of lesions."*
