# Supplementary material for: Incidence and predictors of cardiovascular disease mortality and all-cause mortality in patients with type II diabetes with peripheral arterial disease
Source: PLoS One. 2025 May 16;20(5):e0322502. doi: 10.1371/journal.pone.0322502 (PMC12083800; doi:10.1371/journal.pone.0322502)
Supplement: Attachment — Submitted filename: Response to Reviewers_PONE-D-24-50975R2.docx [file pone.0322502.s004.docx]

**RESPONSES TO REVIEWER'S COMMENTS**

**“Incidence and predictors of cardiovascular disease mortality and all-cause mortality in patients with type II diabetes with peripheral arterial disease**

**# PONE-D-24-50975R2”**

**Shukri AlSaif (Academic Editor)**

**Thank you for submitting your manuscript to PLOS ONE. After careful consideration, we feel that it has merit but does not fully meet PLOS ONE’s publication criteria as it currently stands. Therefore, we invite you to submit a revised version of the manuscript that addresses the points raised during the review process.**

Dear Dr. AlSaif

Thank you for giving us the opportunity to revise and re-submit our manuscript to PLOS ONE.

We extend our sincere gratitude to you and the reviewers for the detailed and constructive feedback. In response, we have made minor revisions to the manuscript as suggested. Please find below our detailed responses to your and the reviewers' comments. In the tracked changes version, revisions in response to the reviewers and editors' comments are highlighted in yellow.

We hope this revised version meets the journal's standards and is now suitable for publication. However, we are open to making any further revisions that may be necessary. We look forward to hearing from you again in due course.

Kind regards,

Kittipan Rerkasem, on behalf of all authors

**REVIEWER #1**

**Thank you for the revision. Unfortunately, there are still several comments. The paper became much better than before. It is minor revision but need to revise.**

Reply: Thank you for your positive feedback and for recognizing the improvements in our revision. We appreciate your detailed review and have carefully addressed the remaining comments to further enhance the clarity and quality of the manuscript.

We have revised the manuscript accordingly, ensuring that all suggested changes have been incorporated. Please find our point-by-point responses below, along with the corresponding revisions in the manuscript.

We sincerely appreciate your time and effort in reviewing our work and believe that these refinements strengthen the overall quality of the paper.

**Minor:**

**1) Acronyms, still need check. T2DM, CVD are spelled out more than twice in the main article. Does Myocardial infarction (MI) need acronym ? Besides, several acronyms such as CKD, COPD are need to spell out. Please check again and also need to check in Tables, Figure legends.**

Reply: Thank you for your careful review and for pointing out the inconsistencies in acronym usage. We have now thoroughly checked the manuscript, including the main text, tables, and figure legends, to ensure that acronyms are introduced only once and spelled out where necessary.

- **T2DM (Type 2 Diabetes Mellitus) and CVD (Cardiovascular Disease)** have been reviewed to ensure they are not spelled out multiple times inappropriately.
- **Myocardial Infarction (MI)** has been checked, and we have ensured that the acronym is introduced properly and used consistently.
- **Chronic Kidney Disease (CKD) and Chronic Obstructive Pulmonary Disease (COPD)** are now spelled out at first mention in both the text and tables/figures.

We appreciate your valuable feedback and believe these revisions improve clarity and consistency in the manuscript.

**2) Table 1 is annoying. why those medications are not only medication name ? (use ? therapy ??) .**

**Also in another Table. Unify such as beta-blocker, antithrombotic, diuretics, statin...**

**dual antiplatelets or single is missing. Don't you have any information about details about it ?**

**ASA, P2Y12, also OACs ??**

**If you combine RAAs inhibitor on Table 4 and 5, why do you separate ACEI and ARB on Table 1.**

Reply: Thank you for your insightful comments regarding Table 1 and the consistency of medication classification across tables. We have carefully revised the tables to improve clarity and standardization based on your suggestions.

- **Medication Naming:** We have revised the presentation of medications to list only the medication names under the category of medication use for clarity and consistency.
- **Antithrombotic:** we have now included a more detailed analysis, specifying dual and single antiplatelet therapy, as well as oral anticoagulants (OACs), to provide a more comprehensive overview.
- **RAAS Inhibitor Consistency:** To maintain uniformity, we have aligned Table 1 with Tables 4 and 5, ensuring that RAAS inhibitors are present consistently.

We appreciate your detailed feedback, which has helped refine the presentation of our data.

**3) what is "S1 Table" ?**

Reply: We apologize for the oversight. "S1 Table" refers to the supplementary table, which was mistakenly omitted in the previous version. We have now added it at the end of the manuscript in the Supporting Data section. Thank you for pointing this out.

**4) In figure legend, intermittent claudication (CL) ?**

Reply: Thank you for pointing this out. We apologize for the misspelling of intermittent claudication (IC) as intermittent claudication (CL). We have corrected this in both the figure and the figure legend to ensure accuracy and consistency.
